# Deep learning meets ontologies: experiments to anchor the cardiovascular disease ontology in the biomedical literature

**DOI:** 10.1186/s13326-018-0181-1

**Published:** 2018-04-12

**Authors:** Mercedes Arguello Casteleiro, George Demetriou, Warren Read, Maria Jesus Fernandez Prieto, Nava Maroto, Diego Maseda Fernandez, Goran Nenadic, Julie Klein, John Keane, Robert Stevens

**Affiliations:** 1 School of Computer Science, University of Manchester, Manchester, UK; 2 Salford Languages, University of Salford, Salford, UK; 3 Departamento de Lingüística Aplicada a la Ciencia y a la Tecnología, Universidad Politécnica de Madrid, Madrid, Spain; 4 Midcheshire Hospital Foundation Trust NHS, Crewe, England UK; 5 Manchester Institute of Biotechnology, University of Manchester, Manchester, UK; 6 Institut National de la Santé et de la Recherche Medicale (INSERM) U1048, Toulouse, France; 7 Universite Toulouse III Paul Sabatier, route de Narbonne, Toulouse, France

**Keywords:** Semantic deep learning, Ontology, Deep learning, CBOW, Skip-gram, Cardiovascular disease ontology, PubMed

## Abstract

**Background:**

Automatic identification of term variants or acceptable alternative free-text terms for gene and protein names from the millions of biomedical publications is a challenging task. Ontologies, such as the Cardiovascular Disease Ontology (CVDO), capture domain knowledge in a computational form and can provide context for gene/protein names as written in the literature. This study investigates: 1) if word embeddings from Deep Learning algorithms can provide a list of term variants for a given gene/protein of interest; and 2) if biological knowledge from the CVDO can improve such a list without modifying the word embeddings created.

**Methods:**

We have manually annotated 105 gene/protein names from 25 PubMed titles/abstracts and mapped them to 79 unique UniProtKB entries corresponding to gene and protein classes from the CVDO. Using more than 14 M PubMed articles (titles and available abstracts), word embeddings were generated with CBOW and Skip-gram. We setup two experiments for a synonym detection task, each with four raters, and 3672 pairs of terms (target term and candidate term) from the word embeddings created. For Experiment I, the target terms for 64 UniProtKB entries were those that appear in the titles/abstracts; Experiment II involves 63 UniProtKB entries and the target terms are a combination of terms from PubMed titles/abstracts with terms (i.e. increased context) from the CVDO protein class expressions and labels.

**Results:**

In Experiment I, Skip-gram finds term variants (full and/or partial) for 89% of the 64 UniProtKB entries, while CBOW finds term variants for 67%. In Experiment II (with the aid of the CVDO), Skip-gram finds term variants for 95% of the 63 UniProtKB entries, while CBOW finds term variants for 78%. Combining the results of both experiments, Skip-gram finds term variants for 97% of the 79 UniProtKB entries, while CBOW finds term variants for 81%.

**Conclusions:**

This study shows performance improvements for both CBOW and Skip-gram on a gene/protein synonym detection task by adding knowledge formalised in the CVDO and without modifying the word embeddings created. Hence, the CVDO supplies context that is effective in inducing term variability for both CBOW and Skip-gram while reducing ambiguity. Skip-gram outperforms CBOW and finds more pertinent term variants for gene/protein names annotated from the scientific literature.

**Electronic supplementary material:**

The online version of this article (10.1186/s13326-018-0181-1) contains supplementary material, which is available to authorized users.

## Background

The sysVASC project [1] seeks to provide a comprehensive *systems medicine* approach to elucidate pathological mechanisms for cardiovascular diseases (CVDs), the number one cause of death globally according to the World Health Organisation [2]. SysVASC developed the CVD ontology (CVDO) to provide the schema to integrate ‘omics data (e.g. genomics, transcriptomics, proteomics and metabolomics) that, together with the most recent scientific papers, are the source of up-to-date knowledge about the biology of the genes and proteins underlying CVD. Extracting knowledge about genes and proteins implicated in CVD for incorporation in the CVDO is an important task in its maintenance. Recognising those genes and proteins within the literature is a required function of this task.

Rebholz-Schuhmann et al. [3] distinguish two approaches to identify gene/protein names from literature:

1. Lexicon based approaches that are based on large terminological resources, e.g. resources generated from large databases like the UniProt Knowledgebase (UniProtKB) [4].
2. Machine Learning (ML) approaches such as conditional random fields [5] that is used in ABNER [6] and BANNER [7].

The first approach has the benefit of normalisation (a.k.a. grounding) [3, 8, 9], i.e. the process of mapping a biological term (e.g. protein name) into a unique entry in a database of biological entities such as UniProtKB. Fundel and Zimmer [10] suggest a limitation that “*the overlap of synonyms in different data sources is rather moderate*” and thus, terms from other databases, such as the HUGO Gene Nomenclature Committee database [11] or Entrez Gene [12], are also needed to develop a more complete lexicon for gene and protein names. Another difficulty is keeping such a lexicon up-to-date, as new term variants for genes and proteins are produced every day [8, 9]. Our study takes the second approach using Deep Learning, an area within ML, to identify suitable term variants (i.e. short forms such as abbreviations or acronyms as well as long forms including phrases) for protein/gene names from the literature.

While conventional ML techniques are limited in their ability to process input data in raw natural language form [13], neural language models from Deep Learning can associate terms with vectors of real-valued features, and semantically related terms end up close in the vector space [13]. The vector representations learnt for the terms are known as word embeddings (i.e. distributed word representations). As the performance of conventional ML techniques are heavily dependent on feature selection [14, 15], a tangible benefit of applying neural language models is that the semantic features of the word embeddings learnt are not explicitly present in the raw natural language input.

This study investigates the suitability of the neural language models CBOW (Continuous Bag-of-Words) and Skip-gram of Mikolov et al. [16, 17] to derive a list of acceptable alternative free-text terms (i.e. term variants) for genes/proteins mentioned in the biomedical literature. The study focuses on two research questions:

1. Is it possible to obtain a list of term variants for a gene/protein from CBOW and Skip-gram word embeddings?
2. Can an improved list of term variants for a gene/protein be produced from the word embeddings by adding knowledge formalised in the CVDO about genes/proteins (i.e. providing more context to reduce ambiguity)?

In this study, a term is a combination of one or more words/tokens, such as “Klf7(−/−)” with one token and “Annexin A4” with two tokens. Terms referring to a gene and its gene product (typically a protein) are likely to appear together as well as separately in the literature. CBOW and Skip-gram use content windows as context, i.e. terms appearing together in the textual input. According to Mikolov et al. [17], CBOW predicts the current term based on the context, while Skip-gram predicts surrounding terms given the current term.

The CVDO represents information about genes and protein from the UniProtKB as a subClassOf axioms (i.e. class expressions or descriptions). With the aid of the CVDO ontology, we expect to obtain terms that provide a more pertinent context to terms from the word embeddings, by, for example a) navigating the class expressions and retrieving the protein name (e.g. ETS translocation variant 1) for a gene symbol (e.g. ETV1); or b) retrieving the full protein name (e.g. Annexin A4) from a partial match (e.g. annexin 4) with the protein class name.

Knowledge within ontologies has been used in two studies – Pilehvar and Collier [18] and Minarro-Gimenez et al. [19] – to assess the quality of word embeddings induced from the literature. As far as we are aware, the use of ontologies per se to provide more context (i.e. extra terms) and improve the list of candidate terms from the word embeddings has not been investigated. This study intends to exploit the relationship between genes and proteins formally represented within the CVDO. A difference between our work and Pilehvar and Collier’s work [18] is that the word embeddings are not modified, i.e. no post-processing of the term vectors is performed. Hence, the use of terms that exploits biological knowledge from the CVDO ontology can be seen as an external intervention.

### Related work

ML methods learn input-output relations from examples with the goal of interpreting new inputs; hence, their performance is heavily dependent on the choice of data representation (or features) to which they are applied [20]. Various types of models have been proposed to represent words as continuous vectors to estimate continuous representation of words and create distributional semantic models (DSMs). DSMs derive representations for words in such a way that words occurring in similar contexts have similar representations, and therefore, the context needs to be defined.

Traditional DSMs include Latent Semantic Analysis (LSA) [21], that generally takes an entire document as a context (i.e. word-document models), and Hyperspace Analog to Language (HAL), [22] that takes a sliding word window as a context (i.e. sliding window models). Random Indexing [23] has emerged as a promising alternative to LSA. LSA, HAL and Random Indexing are spatially motivated DSMs. Examples of probabilistic DSMs are Probabilistic LSA (PLSA) [24] and Latent Dirichlet Allocation (LDA) [25]. While spatial DSMs compare terms using distance metrics in high-dimensional space [26], probabilistic DSMs such as LDA or PLSA measure similarity between terms according to the degree to which they share the same topic distributions [26]. Most DSMs have high computational and storage costs associated with building the model or modifying it due to the huge number of dimensions involved when a large corpus is modelled [26].

This study applies neural language models, i.e. distributed representation of words learnt by neural networks (NNs). Although neural models are not new in DSMs, recent advances in NNs make feasible the derivation of words from corpora of billions of words, hence the growing interest in Deep Learning and the neural language models CBOW and Skip-gram [16, 17]. CBOW and Skip-gram have gained popularity to the point of being the baseline for benchmarking word embeddings [27] and as baseline models for performance comparisons [28]. CBOW and Skip-gram have already been trained to produce high-quality word embeddings from English Wikipedia [27, 29].

Pyysalo et al. [30] and Minarro-Gimenez et al. [19] were the first to apply neural language models to PubMed corpora. Pyysalo et al. [30] used Skip-gram with 22 M PubMed articles as well as more than 672 K PubMed Central Open Access full text articles. The main aim of Pyysalo et al.’s work was to make available word representations (1- to 5-grams) from the literature that could be reused. Minarro-Gimenez et al. [19] used smaller datasets from PubMed as well as from other medical (i.e. Merck Manuals [31], Medscape [32]) and non-medical sources (i.e. Wikipedia [33]). Many later studies have created word embeddings with CBOW and Skip-gram using PubMed corpora.

We describe some of these studies taking into account four tasks that focus on text words, concepts and their relations. At the end of this subsection, we include studies that combine ontologies with word embeddings.

#### Semantic similarity and relatedness task

Pedersen et al. [34] align with more recent studies (Hill et al. [35] and Pakhomov et al. [36]) in emphasising the difference between semantic similarity and semantically relatedness. Pedersen et al. [34] state: “*semantically similar concepts are deemed to be related on the basis of their likeness*”. Both Pedersen et al. [34] and Hill et al. [35] agree with the view of Resnik [37] that “*semantic similarity represents a special case of semantic relatedness*”. Pedersen et al. [34] advocate semantic similarity measures based on is-a relations, where concepts within a hierarchy are linked directly or indirectly. Prior to Pedersen et al. [34], Caviedes and Cimino [38] investigated conceptual similarity metrics based on the minimum number of parent links between concepts. Studies by Caviedes and Cimino [38], Pedersen et al. [34], Hill et al. [35] and Pakhomov et al. [36] made available their datasets of word-pairs together with human judgments of relatedness/similarity. Hill et al.’s [35] dataset of 999 word-pairs, like the WordSimilarity-353 Test Collection [39] (353 word-pairs) and the MEN Test Collection [40] (3 K word-pairs), are common English words. These datasets can be regarded as gold standards for the evaluation of semantic models.

Muneeb [41] et al. applied Skip-gram and CBOW to 1.25 M PubMed articles and evaluated the quality of the word embeddings using the Pedersen et al. [34] word-pairs. Muneeb [41] et al. concluded that Skip-gram is better suited than CBOW for semantic similarity and relatedness. Chiu et al. [42] used the Pyysalo et al. [30] datasets and more than 10 M PubMed English abstracts from the BioASQ challenge [43] for an intrinsic evaluation of the Skip-gram and CBOW word embeddings with the Pakhomov et al. [36] word-pairs. Chiu et al. [42] conclude that Skip-gram shows overall better results for semantic similarity and relatedness than CBOW with different pre-processing.

#### Synonymy detection task

Hill et al. [35] interpret “*relatedness*” as “*association*” and the strongest similarity relation is synonymy. Two well-known datasets for evaluating synonymy are the 80 TOEFL (Test of English as a Foreign Language) synonym questions from [21] and the 50 ESL (English as a Second Language) synonym questions from [44]. Both studies [21] and [44] consist of synonym questions with 4 options that require knowledge of common English words. It should be noted that the TOEFL synonym questions dataset is used in the paper that introduces LSA [21].

To the best of our knowledge no gold standard of word-pairs together with human judgments for synonymy detection exists specific to the biomedical domain.

#### Name entity recognition (NER) and relation extraction tasks

The BioCreative (Critical Assessment of Information Extraction systems in Biology) challenge [45] focuses on recognition of entities in text (i.e. NER) as well as relation extraction. For BioCreative II, Smith et al. [46] mention three tasks: gene mention (GM), gene normalisation (GN), and protein-protein interaction (PPI); the first two are within the scope of NER, whilst the third is a relation extraction task that has NER as a subtask [47].

Pyysalo et al. [30] used Skip-gram to create word embeddings from three datasets: one based on all 22 M PubMed articles; a second based on more than 672 K PubMed Central Open Access full text articles; and a third combining the previous two. Pyysalo et al. [30] clustered the word embeddings created using the well-known K-means clustering algorithm [48] with k = 100. Pyysalo et al. [30] performed a set of NER experiments to assess the quality of both the word embeddings and the clusters created. The NER experiments rely on three biomedical domain corpora: GM using the BioCreative II dataset; anatomical entity recognition using the *Anatomical Entity Mention* corpus [49]; and disease recognition using the NCBI (National Center for Biotechnology Information) *Disease corpus* [50]. More recently Chiu et al. [42] performed an extrinsic evaluation of word embeddings created from CBOW and Skip-gram for NER using two biomedical domain corpora: GM using the BioCreative II dataset and the JNLPBA challenge corpus from Kim et al. [51]. The JNLPBA challenge is a NER task using an extended version of the GENIA corpus (version 3.02) [52]. The GENIA corpus is a manually annotated corpus of 2 K PubMed/MEDLINE abstracts selected from a search using *Medical subject headings* (MeSH) [53] terms “*human*”, “*blood cells*”, and “*transcription factors*”. Chiu et al. [42] conclude that overall Skip-gram shows better results for NER using the datasets from [46, 51] than CBOW with different pre-processing.

Li et al. [54] used Skip-gram with 5.33 M PubMed abstracts obtained from a search with “*protein*” as the keyword. Li et al. [54] like Pyysalo et al. [30] applied the K-means clustering algorithm to cluster word vectors. A difference to the Pyysalo et al. [30] study is that Li et al. [54] employed the Brown tree-building algorithm [55], which is intended for n-gram language models, after applying K-means clustering. To evaluate the PPI extraction performed, Li et al. [54] relied on five publically annotated corpora that has been quantitatively analysed previously in a study by Pyysalo et al. [56].

#### Text categorisation (a.k.a. text classification, or topic spotting)

Sebastiani [15] states that text categorization is "*the activity of labeling natural language texts with thematic categories from a predefined set*". Therefore, assigning keywords or key phrases from MeSH to PubMed/MEDLINE titles or titles-plus-abstracts is a type of text categorisation known as MeSH indexing. The 2017 BioASQ challenge comprised three tasks, one is MeSH indexing, i.e. requesting participants to classify new PubMed articles before curators manually assign MeSH terms to them with some help from the Medical Text Indexer (MTI) [57] from NLM. The MeSHLabeler is an algorithm for MeSH indexing (Liu et al... [58]) that outperforms MTI and won the BioASQ challenge for MeSH indexing in years 2 and 3 of the competition. Both MTI and the MeSHLabeler [58] employ classic bag-of-words representations.

Peng et al [59] used more than 1 M PubMed citations (some downloaded from NLM and some from the BioASQ Year 3 challenge) and introduced DeepMeSH, a workflow that exploits CBOW and obtained a slightly better performance (2% higher micro F-measure) than the MeSHLabeler. It should be noted that MTI, MeSHLabeler, and DeepMeSH employed implementations of the k-nearest neighbour algorithm.

#### Word embeddings and ontologies

The neural language models CBOW and Skip-gram represent each term as a *d*-dimensional vector of *d* real numbers. Taking the vector for a target term and applying cosine similarity, a list of top ranked terms (highest cosine value) can be obtained from the created word embeddings. Minarro-Gimenez et al. [19] and Pilehvar and Collier [18] employed the knowledge represented within ontologies together with metrics based on cosine similarity to evaluate the quality of generated word embeddings. We overview the studies as follows:

1. Minarro-Gimenez et al. [19] focused on four relationships (*may_treat*; *may_prevent*; *has_PE*; and *has_MoA*) from the National Drug File - Reference Terminology (NDF-RT) ontology [60] to assess the word embeddings created based on the hit rate (a.k.a. true positive rate or recall). For example, the number of diseases in a “*may_treat*” relationship with a drug. The hit rate increases if more words for pertinent diseases are within the list of top ranked terms from the word embeddings. Hence, the authors assessed the word embeddings based on a relation extraction task and benchmark against knowledge within the NDF-RT ontology. This early study reported a relatively low hit rate; in contrast, later studies (e.g. Levy et al. [29] and Chiu et al. [42]) benefit from the effect of various hyperparameter configurations.
2. Pilehvar and Collier [18] used the Human Phenotype Ontology (HPO) [61] to assess word embeddings created with Skip-gram from 4B tokens from PubMed abstracts based on two tasks: synonym (alternative names to a class name) and hypernym (X is-a subclass of Y) identification. For the synonym task, the authors benchmark against knowledge within the HPO for two annotation properties; *oboInOwl:hasRelatedSynonym* and *oboInOwl:hasExactSynonym*. For the HPO in OWL, a class name (*rdfs:label*) may have synonyms represented by these two OWL annotation properties. Based on the position in the list of retrieved terms, Pilehvar and Collier [18] calculated the mean and median rank as well as the percentage of phenotypes (i.e. class names in the HPO) for which the rank was equal to one (i.e. the first term in the list retrieved has a synonym in the HPO). Pilehvar and Collier [18] reported improvements by post-processing, i.e. recalculating each dimension of the resulting word vector per phenotype considering a list of weighted words obtained via Babelfy [62]. The authors state that for the phenotype “flexion contracture of digit” a list of 1.3 K weighted words was obtained via Babelfy.

## Methods

This section starts by introducing the three data resources used in two experiments. Next, we describe the two experiments for a gene/protein synonym detection task that use the same vector representations learnt for the terms (i.e. the word embeddings) with CBOW and Skip-gram. As in the synonym detection task described by Baroni et al. [63], both experiments consist of a pair of terms (the target and the candidate) where the cosines (the normalized dot product) of each candidate term vector with the target term vector is computed. Finally, we present the human evaluation performed and the three metrics applied to assess the performance of CBOW and Skip-gram in the gene/protein synonym detection task.

### Data resources

#### Creation of a small-annotated corpus of gene/protein names from 25 PubMed articles

The sysVASC project performed a systematic literature review that involved a PubMed query with the text: “*coronary heart disease AND (proteomics OR proteome OR transcriptomics OR transcriptome OR metabolomics OR metabolome OR omics)*” [Julie Klein 2016, personal communication, 07 June]. The sysVASC review formed part of the data collection protocol to obtain patients with chronic and stable vascular (coronary) disease with exclusion of datasets on acute vascular events or history of potentially interfering concomitant disease. A collection of 34 ‘omics studies/articles with different biological entities of interest (gene, protein, metabolite, miRNA) fulfilled the eligibility criteria. To create a small-annotated corpus relevant for sysVASC and useful for the synonym detection task, we selected 25 of these ‘omics studies that focuses mainly on genes/proteins and are available in the MEDLINE/PubMed database [64]. We left out articles that focus on metabolites or miRNA. The 25 PubMed articles selected were published between 2004 and 2014.

To find the genes/proteins mentioned within the 25 PubMed titles/abstracts, we followed Jensen et al. [65] who divided the task into two: “*first, the recognition of words that refer to entities and second, the unique identification of the entities in question*”. One curator manually annotated 105 terms related to gene/protein names from the 25 PubMed abstracts and titles. Corpus annotation requires at least two annotators and the development of annotation instructions, and thus, the small-annotated corpus cannot be considered a gold standard corpus as only one curator annotated the gene/protein names and no detailed annotation guidelines were developed. For unique identification of genes/proteins we use UniProtKB identifiers. In the UniProtKB each protein entry has two identifiers [66]: 1) an accession number (AC) that is assigned to each amino acid sequence upon inclusion into the UniProtKB; and 2) the “Entry name” (a.k.a. ID), which often contains biologically relevant information. Table 1 contains examples of the manual annotation and normalisation process performed; Table 1 illustrates the lack of standardisation for protein names in the literature.

**Table 1:** *Exemplifying the identification of genes/proteins mentioned within the* 25 *PubMed titles/abstracts*: Terms from PubMed abstract/title from the small-annotated corpus (first column) mapped to UniProtKB ACs (second column) and their corresponding values for skos:altLabel annotation properties of the PxO protein classes (third column)

**Term(s) from PubMed abstract/title**	**UniProtKB AC**	**skos:altLabel for PxO protein classes**
α(1)-antitrypsin alpha-1-antitrypsin	P01009	SERPINA1 (P01009; A1AT_HUMAN) Alpha-1-antitrypsin
annexin 4	P09525	ANXA4 (P09525; ANXA4_HUMAN) Annexin A4
superoxide dismutase 3	P08294	SOD3 (P08294; SODE_HUMAN) Extracellular superoxide dismutase [Cu-Zn]
OLR1	P78380	OLR1 (P78380; OLR1_HUMAN) Oxidized low-density lipoprotein receptor 1
glutathione transferase	P30711	GSTT1 (P30711; GSTT1_HUMAN) Glutathione S-transferase theta-1
FJX1	Q86VR8	FJX1 (Q86VR8; FJX1_HUMAN) Four-jointed box protein 1

The next two examples illustrate the subtask of assigning UniProtKB identifiers to the genes/proteins annotated within the 25 PubMed articles corpus:

- In the abstract of the PubMed article with ID = 15,249,501 the term “heat shock protein-27 (HSP27)” is recognised as a gene/protein name, and subsequently mapped to UniProtKB AC = P04792.
- In the abstract of the PubMed article with ID = 21,938,407 the term “heat shock protein 70 KDa” is recognised as a gene/protein name, and subsequently mapped to UniProtKB AC = P08107. However, on the 27th May, 2015 this UniProtKB entry became obsolete (see [67]), and it is now found with the UniProtKB AC equals P0DMV8 and P0DMV9. Therefore, the term “heat shock protein 70 KDa” is mapped to both UniProtKB ACs, i.e. P0DMV8 and P0DMV9. This example can be seen as a case where some level of ambiguity remains, i.e. more than one UniProtKB AC is assigned to the gene/protein term manually annotated.

The current study is limited to 25 PubMed titles and abstracts, so we acknowledge that some level of ambiguity may remain. We also acknowledge that one straightforward way to disambiguate is by reading the full paper to find the extra information that may aid in uniquely identifying the gene/protein of interest. For example, considering the full text of the article with PubMed ID = 21,938,407, it is clear that the term “heat shock protein 70 KDa” refers to the protein name “Heat shock 70 kDa protein 1A” that has the UniProtKB AC = P0DMV8. Thus, the full article helps to clarify the ambiguity.

The auxiliary file TermsMapped.xls contains the details of the normalisation performed, i.e. the correlation of the 105 terms annotated to the 79 unique UniProtKB entries, where both the UniProtKB identifiers AC and ID are shown.

#### The cardiovascular disease ontology (CVDO)

CVDO provides the schema to integrate the ‘omics data from multiple biological resources, such as the UniProtKB, the miRBase [68] from EMBL-EBI, the *Human Metabolome Database* [69] and the data gathered from various scientific publications (e.g. 34 full-paper ‘omics studies from the sysVASC systematic review and their auxiliary files).

At the core of CVDO is the *Ontology for Biomedical Investigations* [70] along with other reference ontologies produced by the OBO Consortium, such as the *Protein Ontology* (PRO) [71], the *Sequence Ontology* (SO) [72], the three *Gene Ontology* (GO) sub-ontologies [73], the *Chemical Entities of Biological Interest Ontology* [74], the *Cell Ontology* [75], the *Uber Anatomy Ontology* [76], the *Phenotypic Quality Ontology* [77], and the *Relationship Ontology* [78].

For a protein, the CVDO takes as its IRIs the PRO IRIs while also keeping the UniProtKB entry identifiers (i.e. the AC and ID) by means of annotation properties. UniProtKB entry updates could mean changes in the amino acid sequence and/or changes in the GO annotations. The CVDO represents formally the associations between a protein class and classes from the three GO sub-ontologies. In the CVDO there are 172,121 UniProtKB protein classes related to human, and 86,792 UniProtKB protein classes related to mouse. Taking into account the GO annotations for a protein, so far, a total of only 8,196 UniProtKB protein classes from mouse and human have been identified as of potential interest to sysVASC.

**Fig. 1:**
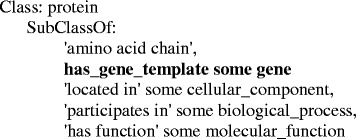
The SubClassOf axioms for the PxO protein class in OWL Manchester Syntax

The CVDO incorporates information about genes and proteins from the UniProtKB, where no alternative names for genes and proteins are available in the UniProtKB downloadable files [79]. In terms of knowledge modelling, the CVDO shares the protein/gene representation used in the Proteasix Ontology (PxO) [80]. The SubClassOf axioms for the PxO protein class in *OWL Manchester Syntax* [81] are shown in Fig. 1. The axiom “*protein SubClassOf (has_gene_template some gene)*” is a class expression that conveys an existential restriction over the object property “*has_gene_template*” from the PRO, where the class “*protein*” (PR:000000001) is from the PRO and the class “*gene*” (SO:0000704) is from the SO. Hence, in the CVDO, as in the PxO, the association between a gene and a protein (gene product) is formally represented with the axiom pattern “*protein SubClassOf (has_gene_template some gene)*” and this is the key knowledge along with the protein and gene names (i.e. lexical content) that we propose to exploit to provide more context for the target terms in Experiment II (see subsection ‘Setup of Experiment I and Experiment II for a gene/protein synonym detection task’ for details).

For a CVDO protein class, we can use its UniProtKB identifier (i.e. AC or ID) to build SPARQL [82] SELECT queries to retrieve: a) the protein class label; and b) the gene class associated to the protein class by exploiting the axiom pattern “*protein SubClassOf (has_gene_template some gene)*”. The auxiliary file TermsMapped.xls contains the gene and protein class labels (i.e. *rdfs:label*) from the CVDO for each of the 79 UniProtKB entries that are part of the small-annotated corpus created.

In the PxO, the annotation property *skos:altLabel* from the Simple Knowledge Organization System (SKOS) [83] is assigned to each protein class that represents a UniProtKB entry. The string value for this annotation property also contains the identifiers (UniProtKB AC and ID) that pinpoint the protein uniquely and has typically the format “*gene symbol (UniProtKB AC; UniProtKB ID) protein name*”. Hence, in the PxO, the association between a protein and a gene is modelled at the logical level with a SubClassOf axiom as well as information attached to the protein class (UniProtKB entry) with no effect on the logical aspects of the class. Table 1 shows how the PxO can provide more context for the terms annotated, e.g. “SERPINA1” is the gene symbol for the protein name “Alpha-1-antitrypsin”.

#### The 14 M PubMed dataset

We downloaded the MEDLINE/PubMed baseline files for 2015 and the up-to-date files through 8th June 2016. To transform the XML PubMed files (see [84] for details of the XML data elements) into a corpus of suitable textual input for Skip-gram and CBOW, two pre-processing steps are carried out. For the first step, we created a processing pipeline that uses open-source software in Python, such as Beautiful soup [85] and the open-source Natural Language Toolkit (NLTK) [86].

When pre-processing the textual input for CBOW and Skip-gram, it is common practice to transform the text into lower-case and to remove systematically all numbers and punctuation marks. This is, however, unsuitable when dealing with protein/gene nomenclature and critical information will be lost if this practice is followed. Tanabe et al. [87] highlight “*gene and protein names often contain hyphens, parentheses, brackets, and other types of punctuation*”. Furthermore, capitalisation and numerals are essential features in symbols or abbreviations. For instance, for human, non-human primates, chickens, and domestic species, gene symbols contain three to six alphanumeric characters that are all in uppercase (e.g. OLR1), while for mice and rats the first letter alone is in uppercase (e.g. Olr1). We therefore decided to alter the commonly employed pre-processing workflow. The Python processing examines the PubMed XML files, locates the data elements of interest and extracts information contained within them while preserving uppercase and punctuation marks within a sentence as well as numbers.

For the second step, we employed word2phrase within the word2vec software package [88] to get n-grams. The title and abstract (if available) of each PubMed publication are the basis to build the DSMs using Skip-gram and CBOW.

Meaningful biomedical terms are typically multi-words; therefore, to obtain better performance titles/abstracts need to be transformed into n-grams. To indicate that more than one word and/or numbers are part of a term, white space is replaced by ‘_’ indicating that the multiple words (and/or numbers) constitute a term.

Once pre-processing is complete, we have a biomedical unannotated corpus of 14,056,762 PubMed publications (titles and available abstracts) with dates of publication between 2000 and 2016 (termed PubMed 14 M for short). The complete list of PubMed IDs can be downloaded from [89].

### Setup of two experiments for a gene/protein synonym detection task

This subsection starts by detailing the creation of the word embeddings with CBOW and Skip-gram using the 14 M PubMed dataset. Next, we detail the setup of two experiments using a small-annotated corpus of gene/protein names and we also specify the exact contribution of the CVDO in Experiment II.

#### Creation of word embeddings with CBOW and Skip-gram

From CBOW and Skip-gram we typically obtain: 1) a lexicon (i.e. a list of terms) in textual format that is constructed from the input data; and 2) the vector representations learnt for the terms, i.e. the word embeddings.

The basic Skip-gram formulation uses the softmax function [17]. The hierarchical softmax is a computationally efficient approximation of the full softmax. If W is the number of words in the lexicon, hierarchical softmax only needs to evaluate about log2(W) output nodes to obtain the probability distribution, instead of needing to evaluate W output nodes. This study uses hierarchical softmax.

In traditional distributional methods, there are a small number of variables known as the hyperparameters of the model. For example, the parameters for the Dirichlet priors in an LDA model are often referred to as hyperparameters. Levy et al. [29] acknowledges that some hyperparameters are tuneable, while others are already tuned to some extent by the algorithms’ designers.

Levy et al. [29] distinguish three types of hyperparameters: 1) pre-processing hyperparameters, 2) association metric hyperparameters, and 3) post-processing hyperparameters. As this study does not modify the resulting term vectors, we present the setup of the pre-processing and association metric hyperparameters implemented in word2vec. We refer the reader to Levy et al. [29] and Chiu et al. [42] that study in detail the effect of various hyperparameter configurations.

Four pre-processing hyperparameters need to be considered:

- *Vector dimension* – In word2vec the default value is 100. We setup the dimensional representation of terms to 300. This value is much lower than Levy et al. [29] that uses 500.
- *Context window size* – In word2vec the default value is 5. We setup the window size to 10, similarly to Levy et al. [29]. word2vec implements a weighting scheme where a size-10 window weights its contexts by 10/ 9, 10/ 10, …, 2/ 1, 10/ 10.
- *Subsampling* – This method dilutes very frequent words [29]. As recommended by Mikolov et al. [17], and like Levy et al. [29], we use the value 1e-5. In word2vec subsampling happens before the textual input is processed and a value zero means that subsampling is switched off.
- *Minimum count (min-count)* – Terms that occur only a few times can be discarded and consequently some terms will not have vector representations. In word2vec the default value of *min-count* is 5, which is the value taken in this study. Chiu et al. [42] show that this hyperparameter has a small effect on performance.

The two association metric hyperparameters are:

- *Negative sampling* – In word2vec by default negative sampling is zero (i.e. not used). However, Skip-gram with negative sampling is acknowledged to provide state-of-the-art results on various linguistic tasks [29]. A higher negative sampling means [29]: a) more data and better estimation; and b) negative examples are more probable. This study does not use negative sampling, and therefore, performance gains for Skip-gram should be relatively easy to obtain if negative sampling is also applied. In other words, it can be argued that by no using negative sampling we are reducing the performance for Skip-gram.
- *Learning rate* – This is a smoothing technique. In word2vec the default value of *alpha* is 0.025, which is used in this study.

In this study to create word embeddings with Skip-gram and CBOW, we use a Supermicro with 256GB RAM and two CPUs Intel Xeon E5–2630 v4 at 2.20GHz. For the 14 M PubMed dataset execution time is less than 1 hour for CBOW and more than 10 hours for Skip-gram.

#### Setup of experiment I and experiment II for a gene/protein synonym detection task

In the small-annotated corpus with 105 terms mapped to 79 UniProtKB entries, not all the UniProtKB entries have the same number of terms manually annotated from the 25 PubMed titles and abstracts. Considering the origin of the target terms and driven by a pragmatic approach, the 79 UniProtKB AC are divided into two sets that participate in each experiment as follows:

- *Experiment I*: the UniProtKB entries that participate in this experiment typically have gene/protein terms manually annotated from the PubMed titles/abstracts. The target terms for this experiment are only gene/protein terms manually annotated with vector representations.
- *Experiment II*: the UniProtKB entries that participate in this experiment typically have gene/protein terms manually annotated from the PubMed titles/abstracts for which there is not a vector representation and/or the CVDO can provide more biological knowledge (e.g. the gene symbol does not appear among the terms manually annotated for the protein/gene of interest). The target terms for this experiment are a combination of: a) gene/protein terms manually annotated from PubMed titles and/or abstracts, and b) terms taken from the CVDO protein and gene class labels. The terms from the CVDO can provide more context to the terms manually annotated to take full advantage of the biological knowledge represented within the CVDO.

The list of acceptable alternative free-text terms (i.e. candidate terms) for genes/proteins is made of terms from the word embeddings with the largest cosine value (the normalized dot product of two vectors) with the target term. In this study, we limit the list to the twelve candidate terms with the highest cosine value (i.e. the top twelve ranked) and we give more importance to the three candidate terms with the highest cosine value (i.e. the top three ranked) within the list. We based our decision in cognitive theories such as that of Novak and Cañas [90] that states “*if we give learners* 10–12 *familiar but unrelated words to memorize in a few seconds, most will recall only* 5–9 *words. If the words are unfamiliar, such as technical terms introduced for the first time, the learner may do well to recall correctly two or three of these. Conversely, if the words are familiar and can be related to knowledge the learner has in her/his cognitive structure,* e.g. *months of the year,* 12 *or more may be easily recalled*”.

Taking into account the word embeddings obtained, the final setup of both experiments is as follows:

- *Experiment I*: this experiment involves 64 UniProtKB entries and 85 target terms, where typically multiple target terms were tried for the same UniProtKB entry. For each target term, a list of the top twelve ranked candidate terms (highest cosine similarity) is obtained from the word embeddings, and thus, this experiment has 1020 pairs of terms (the target and the candidate) to be assessed by the four raters with CBOW and Skip-gram.
- *Experiment II*: this experiment involves 63 UniProtKB entries and 68 target terms, where the correspondence between target terms and UniProtKB entries is almost one-to-one. For each target term, a list of the top twelve ranked candidate terms (highest cosine similarity) is obtained from the word embeddings, and thus, this experiment has 816 pairs of terms (the target and the candidate) to be assessed by the four raters with CBOW and Skip-gram.

A total of 48 UniProtKB entries participate in both Experiment I and II. In Experiment I there are 16 UniProtKB entries that do not participate in Experiment II, for those that the CVDO cannot provide much more added value as they already have the protein name or the protein name and the gene symbol. In Experiment II there are 15 UniProtKB entries that do not participate in Experiment I, those typically correspond to terms annotated from PubMed title/abstracts that do not have a vector and for which the CVDO may supply target terms for them by taking terms from the CVDO protein class expressions and labels.

To clarify the similarities and differences between the two experiments as well as the exact contribution of CVDO in Experiment II, we introduce a simple categorisation that can be applied to: a) the terms from the small-annotated corpus, which appear separated by the character ‘|’ and b) the target terms for the synonym detection task, which appear separated by white space. The simple categorisation introduced consists of five categories:

1. *Only gene symbol* –Term is the gene symbol. For example: OLR1.
2. *Gene symbol appears* – A combination of terms among which the gene symbol appears. An example from the small-annotated corpus is C3|complement C3. An example from the target terms for the synonym detection task is: oxidized_low-density_lipoprotein receptor_ 1 OLR1.
3. *Refer protein name* – Terms that refer to the protein name. An example from the small-annotated corpus is CTRP1|C1q/TNF-related protein 1|adipokine C1q/TNF-related protein (CTRP). An example from the target terms for the synonym detection task is collagen_type_1.
4. *Only protein name* –The exact protein name as it appears in the UniProtKB. An example from the target terms for the synonym detection task is glutathione_S-transferase theta-1.
5. *Terms from protein name* –Terms taken from the protein name as it appears in the UniProtKB. An example from the target terms for the synonym detection task is c1q tumor_necrosis_factor.

Both categories “*Only protein name*” and “*Terms from protein name*” are applied only to the target terms and take into account the protein name as it appears in the UniProtKB, which is the lexical content from protein class labels (i.e. rdfs:label) within the CVDO.

Table 2 for Experiment I and Table 3 for Experiment II apply the simple categorisation proposed to the terms from the small-annotated corpus (first column in the Tables); and to the target terms for the synonym detection task (second column in the Tables). The third column represents the number of target terms. For example, in Table 2 for Experiment I the higher number of target terms corresponds to the category “*Only gene symbol”* with 34 target terms, where 13 of them correspond to terms from the small-annotated corpus belonging to the category “*Gene symbol appears*”.

**Table 2:** *Setup for Experiment I*: The simple categorisation introduced (see ‘Setup of Experiment I and Experiment II for a gene/protein synonym detection task’) has been applied to the terms from PubMed abstract/title from the small-annotated corpus (first column) as well as to the target terms (second column). Each row of the third column contains the number of target terms for the experiment taking into account the categories that appear in the first and second column

**Simple categorisation introduced**		
**Terms from PubMed titles/abstracts**	**Target terms**	**n**
Gene symbol appears	Gene symbol appears	5
Gene symbol appears	Only gene symbol	13
Gene symbol appears	Only protein name	3
Gene symbol appears	Refer protein name	2
Gene symbol appears	Terms from protein name	2
Only gene symbol	Only gene symbol	21
Refer protein name	Gene symbol appears	1
Refer protein name	Only protein name	16
Refer protein name	Refer protein name	18
Refer protein name	Terms from protein name	4

**Table 3:** *Setup for Experiment II and contribution of the CVDO*: The simple categorisation introduced (see ‘Setup of Experiment I and Experiment II for a gene/protein synonym detection task’) has been applied to the terms from PubMed abstract/title from the small-annotated corpus (first column) as well as to the target terms (second column). Each row of the third column contains the number of target terms for the experiment taking into account the categories that appear in the first and second column

Simple categorisation introduced			
Terms from PubMed titles/abstracts	Target terms	n	Terms added by CVDO to the target terms
Gene symbol appears	Gene symbol appears	6	Terms from protein name **(R)**
Gene symbol appears	Only protein name	1	Protein name **(R)**
Gene symbol appears	Refer protein name	1	Terms referring to the protein name **(R)**
Gene symbol appears	Terms from protein name	2	Terms from protein name **(R)**
Only gene symbol	Gene symbol appears	20	Terms from protein name **(R)**
Only gene symbol	Only protein name	4	Protein name **(R)**
Refer protein name	Gene symbol appears	27	Terms from protein name and gene symbol **(R)**
Refer protein name	Only gene symbol	2	Gene symbol **(R)**
Refer protein name	Only protein name	2	Protein name
Refer protein name	Refer protein name	1	Terms referring to the protein name
Refer protein name	Terms from protein name	2	Terms from protein name

The fourth column indicates the terms added by the CVDO, when the symbol **(R)** appears it means that the protein class expressions within the CVDO are used to add terms to the target terms

Table 3 for Experiment II has a fourth column to clearly indicate the origin of the terms added by the CVDO to the target terms. In Table 3 for Experiment II the higher number of target terms corresponds to the category “*Gene symbol appears”* with 53 target terms, where 27 of them correspond to terms from the small-annotated corpus belonging to the category “*Refer protein name*”. For these 27 target terms, the CVDO added terms from protein name and gene symbol, and therefore, exploiting the protein class expressions within the CVDO.

In the rows of the fourth column of Table 3, the symbol **(R)** means that the protein class expressions within the CVDO are used to add terms to the target terms. Hence, 63 of the 68 target terms (i.e. 93%) exploit the relationship between genes and proteins modelled in the CVD ontology. Only 5 target terms (i.e. 7%) exploit lexical content from protein class labels.

### Human evaluation and metrics to assess the performance of Skip-gram and CBOW in experiment I and II

To assess how many free-text candidate terms within the list can be actually considered to be term variants (e.g. synonyms, abbreviations, and variant spellings) we rely on four domain experts to rate pairs of terms (the target and the candidate) and assess whether the candidate term is a *full-term variant* (FTV for short), a *partial-term variant* (PTV for short), or a *non-term variant* (NTV for short, meaning none of the previous categories). The same four raters (A, B, C, and D) assessed the 3672 pairs of terms (target term and candidate term) in Experiments I and II. Raters A and D are trained terminologists who work in biomedicine; Raters B and C are bio-curators, who at the time of the study worked on biochemical knowledge extraction from textual resources.

We established a strict criterion to mark each pair of terms (the target and the candidate) from the CBOW and Skip-gram word embeddings. Following Nenadic et al. [91], a candidate term is marked as FTV only when the term falls within the following types of term variation: a) orthographic, b) morphological, c) lexical, d) structural, or e) acronyms and abbreviations. Considering the biomedical value of phraseological expressions (e.g. “*ankyrin-B_gene*” or “*CBS_deficiency*”), they are marked as PTV if they refer to the same protein/gene of interest.

In order to calculate precision and recall, which are well-known metrics for evaluating retrieval (classification) performance, one set of annotations should be considered as the gold standard [92]. In this study, we advocate a voting system as we have four annotators/raters and two of them are bio-curators. Hence, we do not follow studies like Thompson et al. [93], which calculate precision and recall, and use F score (i.e. a metric that combines precision and recall) as a way of calculating inter-annotator agreement.

When having two raters/coders/annotators, the inter-annotator agreement is typically calculated using Cohen’s Kappa measure [94]. For more than two coders, Fleiss [95] proposed a coefficient of agreement that “*calculates expected agreement based on the cumulative distribution of judgments by all coders*” [96]. This measure of inter-annotator agreement is also known as *Fleiss’s multi-π* as it can be interpreted as a generalisation of *Scott’s π* [97]. It should be noted that when all disagreements are considered equal, as in this study, *Fleiss’s multi-π* is nearly identical to *Krippendorff’s α* [98], which is an agreement coefficient recommended in computational linguistics for coding tasks without involving nominal and disjoint categories [96]. Hence, we adhere to Artstein and Poesio [96] who state that it is better practice in computational linguistics to use generalised versions of the coefficients (e.g. *Fleiss’s multi-π*) instead of measuring agreement separately for each pair of coders (Cohen’s Kappa measure), and then report the mean.

In this study three metrics are used to assess the performance of CBOW and Skip-gram for the synonym detection task. The first metric is the area under the Receiver Operating Characteristics (ROC) curve for a binary classifier. FTV and PTV can be merged into one category called term variant or TV for short. Hence, the multiple class classification problem can be reduced to three binary classification problems: 1) FTV and non-FTV; 2) PTV and non-PTV; and 3) TV and non-TV. This study uses ROC curves instead of precision-recall curves, as ROC curves do not change if the class distribution is different [99]. The second metric is the median of the rank that was used by Pilehvar and Collier [18] in a synonym and hypernym identification tasks with Skip-gram. The third metric is the number of term variants (i.e. FTV and/or PTV) found for each of the 79 UniProtKB entries within the small-annotated corpus of gene/protein names from 25 PubMed articles.

#### Receiver operating characteristics (ROC) curve and the area under the ROC curve (AUC)

To compare classifiers, calculating the area under the ROC curve, the so-called AUC [100–102], is a common method. Fawcett [99] defines the ROC curve as “*a technique for visualizing, organizing and selecting classifiers based on their performance*”. As Bradley [100] states “*when comparing a number of different classification schemes it is often desirable to obtain a single figure as a measure of the classifier's performance*”. The AUC can be interpreted as a probability of correct ranking as estimated by the Wilcoxon statistic [101]. Furthermore, as Hand and Till [102] highlight, the AUC is “*independent of costs, priors, or (consequently) any classification threshold*”.

A ROC curve has two dimensions, where typically *TP rate* is plotted on the Y axis and *FP rate* is plotted on the X axis [99]. *TP rate* stands for *true positive rate* (a.k.a. hit rate or recall or sensitivity) and is the proportion of positives correctly classified as positives; *FP rate* stands for *false positive rate* (a.k.a. false alarm rate) and is the proportion of negatives that are incorrectly classified as positive. For the perfect classifier *TP rate* = 1 and *FP rate* = 0. In the ROC curves, the diagonal line (y = x) is also plotted which represents random guessing [99] and acts as the baseline for ROC. A random classifier typically ‘slides’ back and forth on the diagonal [99].

As the candidate terms evaluated for the human raters are ranked (highest cosine value), we have the category assigned by the rater to each candidate term (FTV, PTV, or NTV) as well as the position that the candidate term has in the top twelve ranked list. Firstly, for each experiment and rater, we created a table with twelve rows and three columns: frequency of FTV, frequency of PTV, and frequency of NTV. For example, the frequency of FTV column accounts for the number of times that a rater assigned FTV for the term in the *i*^*th*^ position in the list, with i = [1,…, 12]. Secondly, we calculated the cumulative frequency, and thus, three more columns were added. The cumulative frequency is calculated in descending order, where the value of the cumulative frequency for the *i*^*th*^ position in the list adds to the value from the frequency column in the *i*^*th*^ position, the value of the cumulative frequency for the (*i-*1*)*^*th*^ position in the list. Thirdly, we calculated the cumulative rate, and therefore, three more columns were added. For example, the cumulative rate of FTV column is calculated by dividing the values of the cumulative frequency of FTV column by the total number of FTV assigned by the rater. Hence, the last value in any of the cumulative rate columns (12th position) is equal to 1. In the ROC curves, we plot the cumulative rates obtained. Hence, the ROC curves for FTV, PTV, and TV end at (1, 1).

The values for the AUC go from zero to one. Random guessing will have an AUC = 0. 5 and “*no realistic classifier should have an AUC less than 0.* 5” [99]. We plot ROC curves for FTV, PTV, and TV and calculate the AUC for each rater and experiment.

#### The median of the rank per human rater

Based on the domain expert category assigned (FTV, PTV, or NTV) to each candidate term from the word embeddings, as well as the position that the candidate term has in the top twelve ranked list (highest cosine similarity), we can calculate the median of the rank for FTV and PTV per rater. A lower median means that the terms marked as terms variants (full or partial) appear at the beginning of the list.

#### Number of UniProtKB ACs and CVDO classes with a term variant

Based on the 79 unique UniProtKB entries from the small-annotated corpus we implement a voting system based on raters’ judgement and determine for how many of the 79 UniProtKB entries mapped to CVDO classes, term variants were found. The voting system takes the domain expert category assigned (FTV, PTV, or NTV) and considers that a candidate term from the top twelve ranked list is an *FTV* if at least one of the four raters assigned the category FTV once. Likewise, and more generally, if at least one of the four raters marks a candidate term from the top twelve ranked list as FTV or PTV, the voting system concludes a *TV* has been found.

## Results

We start by illustrating the results obtained in Experiment I and II with CBOW and Skip-gram. Next we report the human inter-annotator agreement and the results obtained for the three metrics to assess the performance of CBOW and Skip-gram in the gene/protein synonym detection task.

### Exemplifying the results obtained for the gene/protein synonym detection task in experiment I and II

Each auxiliary file - CBOW.xls and Skip-gram.xls - contains the 1836 pairs of terms (target term and candidate term) from the word embeddings created, along with the cosine similarity obtained for each pair of terms. Each file includes the list of the top twelve ranked candidate terms (highest cosine similarity) per target term, where the last four columns have the human judgement (FTV, PTV, or NTV) by the four raters A-D. Each target term: a) relates to a UniProtKB entry that has a UniProtKB identifier (i.e. the UniProtKB AC column) and also a string value for the annotation property *skos:altLabel* for the PxO protein class, b) has a unique identifier in column nQ that also appears in the auxiliary file TermsMapped_votingSystem.xls, c) contains at least one term from the small-annotated corpus (Term from the PubMed titles/abstracts column), and d) participates in Experiment I (abbreviated as Exp I) or Experiment II (abbreviated as Exp II) as indicated in the Experiment column.

We use target terms from the auxiliary files to illustrate the ranked list of the top twelve candidate terms (highest cosine similarity) for gene/protein names obtained from the word embeddings created with CBOW and Skip-gram for Experiments I and II.

Table 4 shows the list of the top twelve candidate terms (highest cosine similarity) obtained with CBOW and Skip-gram word embeddings in Experiment I for the target term “*KLF7*”, which is a gene symbol and appears as such in the abstract of the PubMed article with ID = 23,468,932. For CBOW, all four raters agree that there is not a full or partial gene/protein term variant (i.e. FTV or PTV) among the list of candidate terms; in other words, all the top twelve ranked candidate terms for CBOW were marked as NTV by the four raters. For Skip-gram, all four raters agree that: a) the candidate term in the second position in the list is an FTV, and b) the candidate term in the third position in the list is a PTV. Hence, in Experiment I for the target term “*KLF7*”, CBOW could not find a TV while Skip-gram found an FTV and also a PTV among the top three ranked candidate terms in the list. From a biological point of view, the target term “KLF7” denotes a human gene, while the candidate term in the second position in the list “Klf7” denotes the equivalent gene in mice. The genes KLF7 and Klf7 are orthologs according to the NCBI [103]. The candidate term in the third position in the list “Klf7(−/−)” refers to mice which are homozygous for the Klf7 gene knockout. Hence, the pre-processing of the 14 M PubMed dataset that keeps uppercase, punctuation marks, and numbers, demonstrably preserves valuable biological information.

**Table 4:** *Exemplifying results for Experiment I*: Top twelve ranked candidate terms (highest cosine similarity) from the word embeddings created with CBOW and Skip-gram for the target term “KLF7” that appears in the abstract of the PubMed article with ID = 23,468,932

	CBOW		Skip-gram	
Rank	Candidate terms from word embeddings	Cosine	Candidate terms from word embeddings	Cosine
1	MoKA	0.376371	Prrx2	0.601920
2	pluripotency-associated_genes	0.335113	Klf7	0.592946
3	Sp1_regulates	0.334092	Klf7(−/−)	0.590523
4	LOC101928923	0.333423	RXRG	0.589875
5	p107_dephosphorylation	0.331689	LOC101928923	0.585979
6	PU_1	0.329925	SOX-17	0.585295
7	histone_demethylase	0.323529	rs820336	0.585094
8	gene_promoter	0.321640	GLI-binding_site	0.581073
9	homeobox_protein	0.319997	Tead2	0.580012
10	histone_arginine	0.315875	hHEX	0.579868
11	transfated	0.314202	ACY-957	0.579542
12	are_unable_to_repress	0.313112	ETS1	0.577272

The term “OLR1”, which is a gene symbol, appears as such in the abstract of the PubMed article with ID = 22,738,689. Using “OLR1” as the target term in Experiment I for CBOW and Skip-gram, no candidate terms from the word embeddings were suitable as FTV or PV according to all four raters.

In Experiment II, the term “*oxidized_low-density_lipoprotein receptor_1*” that corresponds to the protein name is added to the gene symbol “OLR1” to create a target term. Table 5 shows the top twelve ranked candidate terms obtained by CBOW and Skip-gram word embeddings in Experiment II using these two terms “*oxidized_low-density_lipoprotein receptor_1 OLR1*” as the target term. Therefore, the target contains a term that exploits knowledge within the CVDO and, more concretely, the association relationship formally represented between genes and proteins. As the CVDO provides more context, in Experiment II with both CBOW and Skip-gram, suitable term variants (FTV as well as PTV) were found for the protein/gene name.

**Table 5:** *Exemplifying results for Experiment II*: Top twelve ranked candidate terms (highest cosine similarity) from the word embeddings created with CBOW and Skip-gram using two terms as target: “OLR1” from the abstract of the PubMed article with ID = 22,738,689; and “oxidized_low-density_lipoprotein receptor_ 1” that is the CVDO protein class name (rdfs:label) for the CVDO class gene with name (rdfs:label) OLR1. Hence, the target term exploits the protein class expressions within the CVDO

	CBOW		Skip-gram	
Rank	Candidate terms from word embeddings	Cosine	Candidate terms from word embeddings	Cosine
1	atherogenesis	0.469405	lectin-like_oxidized_low-density_lipoprotein	0.688603
2	atherosclerosis	0.465861	(LOX-1)_is	0.672042
3	CD36	0.439280	atherosclerosis_we_investigated	0.669050
4	LOX-1	0.424173	receptor-1	0.664891
5	atherosclerotic_lesion_formation	0.416537	lectin-like_oxidized_LDL_receptor-1	0.663988
6	vascular_inflammation	0.414620	lOX-1_is	0.660110
7	inflammatory_genes	0.411186	human_atherosclerotic_lesions	0.657075
8	atherosclerotic_lesions	0.405906	oxidized_low-density_lipoprotein_(ox-LDL)	0.655515
9	monocyte_chemoattractant_protein-1	0.398739	oxidized_low-density_lipoprotein_(oxLDL)	0.654965
10	plaque_destabilization	0.398201	(LOX-1)	0.652099
11	oxidized_low-density_lipoprotein_(oxLDL)	0.397967	proatherosclerotic	0.651571
12	atherosclerosis_atherosclerosis	0.396677	receptor-1_(LOX-1)_is	0.649000

Tables 4 and 5 show higher cosine values for Skip-gram than CBOW. As cosine similarity gives an indication of how strongly semantically related is the pair of terms (the target and the candidate), it seems natural that Skip-grams finds more term variants than CBOW.

Table 6 shows the categories FTV, PTV, or NTV assigned by the four human raters (A-D) to the top twelve ranked candidate terms obtained for Skip-gram in Experiment II using two terms “*oxidized_low-density_lipoprotein receptor_1 OLR1*” as the target term. This list of the top twelve ranked candidate terms appears in the right-hand side of Table 5. The last three columns of the Table exemplify the voting system (abbreviated as VS) applied: full term variant (VS: FTV column), full term variant among the top three (VS: FTV for top three column), and full and/or partial term variant (VS: TV column).

**Table 6:**
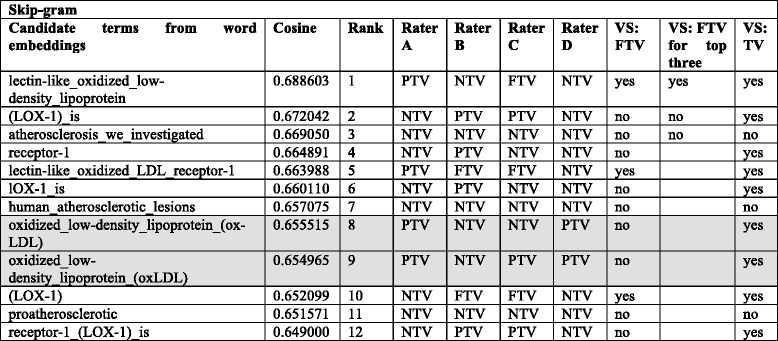
*Exemplifying human judgements and voting system for Skip-gram*: Categories FTV, PTV, or NTV assigned for the four human raters (A, B, C, and D) to the top twelve candidate terms for the target term “oxidized_low-density_lipoprotein receptor_ 1 OLR1” in Experiment II using Skip-gram. The last three columns show the voting system (VS) applied for FTV (full term variant), FTV among the top three, and TV (full and/or partial term variant). The two rows in grey background remark how two almost identical candidate terms from the word embeddings are marked differently by rater C, and thus, the manual annotation by raters is error-prone

Two rows appear with a grey background in Table 6. They indicate the process of manually assigned categories to be error-prone as Rater C assigned NTV to the candidate term in the eighth position in the list “*oxidized_low-density_lipoprotein_(ox-LDL)*” while marking PTV for the candidate term in the ninth position, “*oxidized_low-density_lipoprotein_(oxLDL)*”. From visual inspection, the only difference in these two candidate terms is the appearance of, or lack of, a ‘-‘. It should be noted that Raters A, B, and D mark both candidate terms in the list equally, although they differ in the category assigned. The biological background knowledge of Raters B and C (curators) and their impact on the manual categorisation process can be deduced from Table 6. Gene *OLR1* has a well-known alias LOX-1, and thus, Raters B and C marked the candidate terms as FTV if LOX-1 appears alone or PTV if LOX-1 appears in combination with other term(s); however, Raters A and D marked all the candidate terms as NTV where LOX-1 appears.

### Human evaluation and metrics to assess the performance of Skip-gram and CBOW in Experiment I and II

We start reporting on the inter-annotator agreement coefficients for the four raters. For pairwise inter-annotator agreement (the Cohen’s Kappa measure) per experiment and model, we refer the reader to auxiliary file pairwiseIAA.xls. All the inter-annotator agreement coefficients are calculated with the implementations from the NLTK [86]:

- Using data from auxiliary file CBOW.xls, the *Fleiss’s multi-π* for the four raters in Experiment I is 0.763205 and for Experiment II is 0.730869. The *Krippendorff’s α* for the four raters in Experiment I is 0.763211 and for Experiment II is 0.730874.
- Using data from auxiliary file Skip-gram.xls, the *Fleiss’s multi-π* for the four raters in Experiment I is 0.794919 and for Experiment II is 0.673514. The *Krippendorff’s α* for the four raters in Experiment I is 0.794938 and for Experiment II is 0.674181.

As expected, the values obtained for the *Fleiss’s multi-π* and the *Krippendorff’s α* for the four raters are nearly identical. The inter-annotator agreement is lower for Experiment II, which is more challenging in terms of biological background knowledge. Camon et al. [104] reports that the chance of curator agreement is 39% to 43% when annotating proteins in the UniProtKB with terms from the GO. Hence, inter-annotator agreement from 0.6734 (lowest value for *Fleiss’s multi-π*) to 0.7949 (highest value for *Fleiss’s multi-π*) appears reasonable.

#### Receiver operating characteristics (ROC) curve and the area under the ROC curve (AUC)

**Fig. 2:**
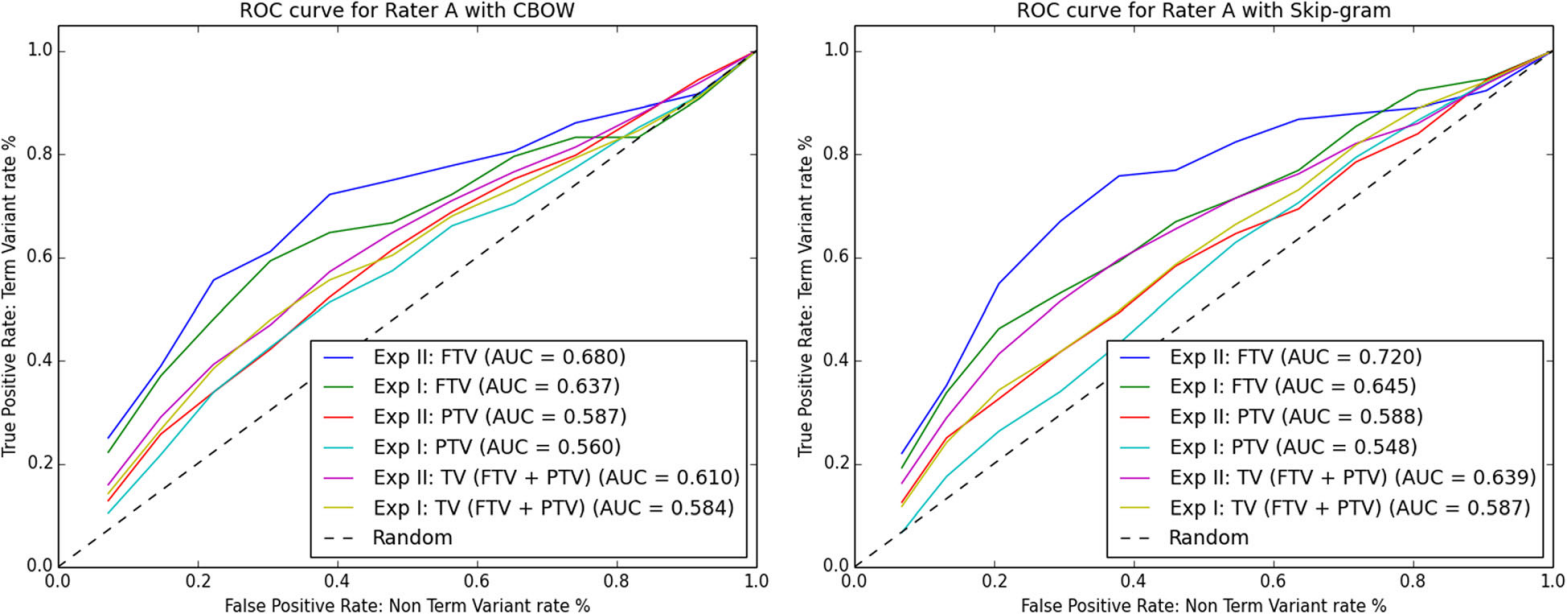
ROC curves for rater A: left-hand side CBOW and right-hand side Skip-gram. Abbreviations: Exp I = Experiment I; Exp II = Experiment II

**Fig. 3:**
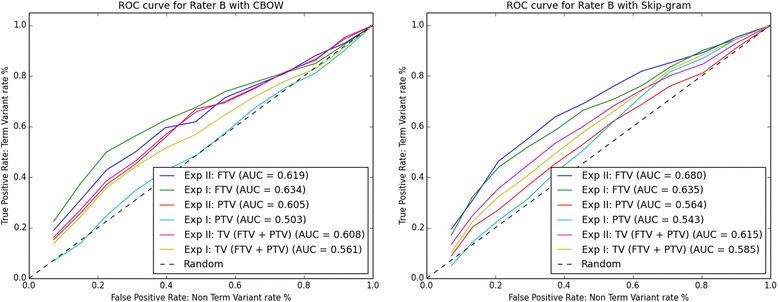
ROC curves for rater B: left-hand side CBOW and right-hand side Skip-gram. Abbreviations: Exp I = Experiment I; Exp II = Experiment II

**Fig. 4:**
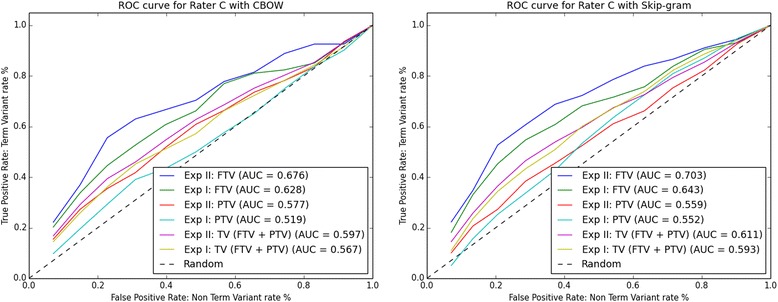
ROC curves for rater C: left-hand side CBOW and right-hand side Skip-gram. Abbreviations: Exp I = Experiment I; Exp II = Experiment II

**Fig. 5:**
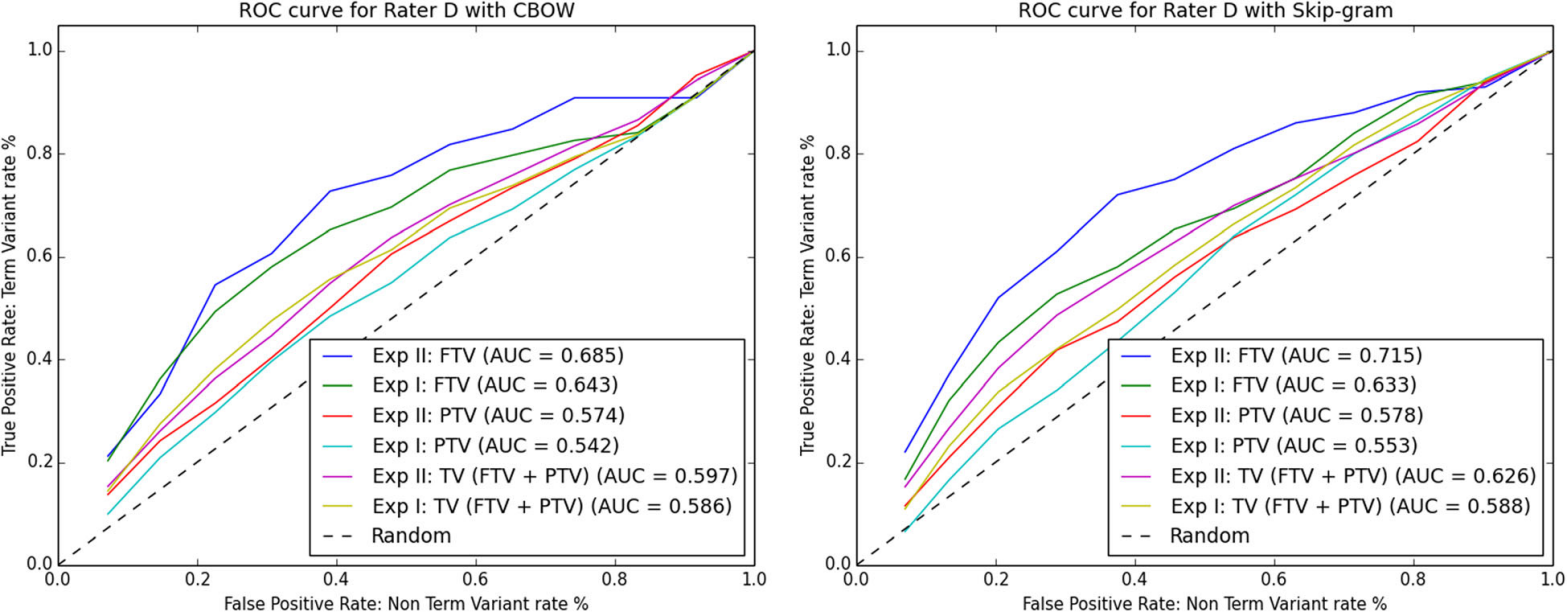
ROC curves for rater D: left-hand side CBOW and right-hand side Skip-gram. Abbreviations: Exp I = Experiment I; Exp II = Experiment II

Using data from auxiliary files CBOW.xls and Skip-gram.xls, we plotted the ROC curves. For each Rater A-D the ROC curves are shown in Figs. 2, 3, 4 and 5 respectively. The ROC curves on the left-hand side plot FTV, PTV, and TV (i.e. the combination of FTV and PTV) for CBOW in Experiment I (abbreviated as Exp I) and Experiment II (abbreviated as Exp II). The ROC curves on the right-hand side plot FTV, PTV, and TV for Skip-gram in Experiment I and II.

Looking at the AUC values for FTV, PTV, and TV in Figs. 2, 3, 4 and 5, it can be observed that for all four raters:

- The AUC values for FTV, PTV, and TV are always greater than 0. 5 (i.e. better than random guessing) for both CBOW and Skip-gram in Experiments I and II.
- The AUC values for TV are always greater in Experiment II than in Experiment I for both CBOW and Skip-gram.
- The AUC values for TV are always greater for Skip-gram than for CBOW in both Experiment I and II.
- The AUC values for PTV are always greater in Experiment II than in Experiment I for both CBOW and Skip-gram.
- The higher AUC values are for FTV with both CBOW and Skip-gram.
- The maximum AUC values are for FTV in Experiment II with Skip-gram.

The only noticeable discrepancy is that for three Raters (A, C, and D), CBOW has the higher AUC values for FTV in Experiment II, and for Rater B the higher AUC value for CBOW is for FTV in Experiment I.

Considering the ROC curves and the AUC values, we conclude that: a) Skip-gram outperforms CBOW in both Experiments for the binary classification problem TV and non-TV, b) both CBOW and Skip-gram perform best for the binary classification problem FTV and non-FTV, c) the best performance is for Skip-gram in Experiment II for the binary classification problem FTV and non-FTV.

#### The median of the rank per human rater

Using data from the auxiliary files CBOW.xls and Skip-gram.xls, we calculated the median of the rank. Table 7 shows the median of the rank for Raters A-D. From Table 7:

- For CBOW and Skip-gram in Experiment II, the mean of the median of the rank for an FTV is 3.
- For CBOW and Skip-gram in Experiment I, the mean of the median of the rank for an FTV is 4.
- For Skip-gram in Experiment I and II, the median of the rank for a PTV is 6 for all four raters.
- For CBOW in Experiment II, the median of the rank for a PTV is 5 for all four raters.
- For CBOW in Experiment I, the mean of the median of the rank for a PTV is 6.

**Table 7:** *Median of the rank for CBOW and Skip-gram in Experiments I and II for each of the four raters*

Experiment	Model	Rater A		Rater B		Rater C		Rater D	
		Median FTV	Median PTV	Median FTV	Median PTV	Median FTV	Median PTV	Median FTV	Median PTV
I	CBOW	4	5	3	7	4	6	4	6
II	CBOW	3	5	4	5	3	5	3	5
I	Skip-gram	4	6	4	6	4	6	4	6
II	Skip-gram	3	6	4	6	3	6	3	6

The higher the rank (i.e. lowest number) for an FTV the better, and thus, results obtained for both CBOW and Skip-gram indicate that CVDO can slightly improve the ranking of an FTV from being among the top four ranked candidate terms in Experiment I (without the aid of the CVDO) to be among the top three ranked candidate terms in Experiment II (with the aid of the CVDO).

#### Number of UniProtKB entries mapped to CVDO gene and protein classes with term variants

The auxiliary file TermsMapped_votingSystem.xls contains the results of the voting system according to the simple categorisation introduced (see subsection ‘Setup of Experiment I and Experiment II for a gene/protein synonym detection task’), which has been applied to the terms from PubMed abstract/title from the small-annotated corpus (Category for terms from the title/abstract column) as well as to the target terms (Category for target terms column). The file has 153 target terms, each with a unique identifier in column nQ that also appears in each auxiliary file under the column nQ. Of these 153 target terms: 85 target terms for 64 UniProtKB entries are mapped to CVDO protein and gene classes in Experiment I (abbreviated as Exp I), and 68 target terms for 63 UniProtKB entries are mapped to CVDO protein and gene classes in Experiment II (abbreviated as Exp II). The last six columns display the presence (i.e. value equals 1) or absence (i.e. value equals 0) for each neural language model CBOW and Skip-gram of: full term variants (i.e. FTV) among the top twelve ranked candidate terms for the target term; FTV among the top three ranked candidate terms for the target term; and term variants (i.e. FTV and/or PTV) among the top twelve ranked candidate terms for the target term.

Tables 8–11 take the data from auxiliary file TermsMapped_votingSystem.xls and summarise the results obtained.

**Table 8:** *Overall performance of CBOW and Skip-gram according to the voting system*: Number of unique UniProtKB entries and number of term pairs for protein/gene names that are involved in Experiment I, II, and combined (i.e. merging Experiment I and II)

				Voting system		
Experiment	Model	Number of terms pairs	Number of UniProtKB entries	Number FTV	Number FTV for top three	Number TV (%)
I	CBOW	1020	64	31	21	43 (67%)
II	CBOW	816	63	29	21	49 (78%)
I and II combined	CBOW	1836	79	47	37	64 (81%)
I	Skip-gram	1020	64	49	37	57 (89%)
II	Skip-gram	816	63	56	51	60 (95%)
I and II combined	Skip-gram	1836	79	71	63	77 (97%)

According to the voting system, for each model the last three columns show: the number of full term variants among the top twelve ranked candidate terms for the UniProtKB entries (Number FTV column); the number of full term variants among the top three ranked candidate terms for the UniProtKB entries (Number FTV for top three); and the number and % of term variants (i.e. FTV and/or PTV) among the top twelve ranked candidate terms for the UniProtKB entries (Number TV column)

Table 8 shows the overall performance of CBOW and Skip-gram in Experiment I and II according to the voting system, which can be summarised as follows:

- In Experiment I, Skip-gram finds term variants among the top twelve ranked candidate terms (Number TV column) for 89% of the 64 unique UniProtKB entries mapped to CVDO gene and protein classes, while CBOW finds term variants for 67%. Hence, using as target terms only terms from PubMed titles/abstracts, the word embeddings generated with the 14 M PubMed dataset can obtain a list of term variants for gene/protein names.
- In Experiment II (with the aid of the CVDO), Skip-gram finds term variants among the top twelve ranked candidate terms (Number TV column) for 95% of the 63 unique UniProtKB entries mapped to CVDO gene and protein classes, while CBOW finds term variants for 78%. Hence, both neural language models Skip-gram and CBOW provide more term variants (FTVs and/or PTVs) if the CVDO is used to provide more context for the target terms, and therefore increasing the chances of finding suitable term variants for a gene/protein name.
- Combining the results of both experiments, Skip-gram finds term variants (FTVs and/or PTVs) among the top twelve ranked candidate terms for 97% of the 79 UniProtKB entries mapped to CVDO gene and protein classes, while CBOW finds term variants for 81%.
- The number of term pairs in Experiment I is 1020 while in Experiment II it is 816, however more term variants are found in Experiment II. Hence, knowledge from the CVDO (i.e. mostly the protein class expressions along with lexical content from protein class labels) to make the term targets more efficient as fewer term pairs are needed to produce more term variants.

Table 9 shows the performance of CBOW and Skip-gram according to the voting system and considers the number of UniProtKB entries that participate in each experiment. The third column contains the number of target terms for the experiment considering the number of UniProtKB entries, where Experiment I has a higher number of target terms per UniProtKB entry than Experiment II. In Table 9 there are some rows with a grey background; they refer to the 48 UniProtKB entries that participate in both Experiments. There are 16 UniProtKB entries that participate only in Experiment I and 15 UniProtKB entries that participate only in Experiment II. Considering each number of UniProtKB entries in an Experiment, it can be observed that Skip-gram always outperforms CBOW and finds more FTVs among the top twelve ranked candidate terms (Number FTV column); FTVs among the top three ranked candidate terms (Number FTV for the top three column); and TVs among the top twelve ranked candidate terms (Number TV column). By considering only the 48 UniProtKB entries that participate in both Experiments, it can be observed that:

- CBOW finds TVs (Number TV column) among the top twelve ranked candidate terms for 30 of the 48 UniProtKB entries in Experiment I (i.e. 62%) and for 37 of the 48 UniProtKB entries in Experiment II (i.e. 77%).
- Skip-gram finds TVs (Number TV column) among the top twelve ranked candidate terms for 42 of the 48 UniProtKB entries in Experiment I (i.e. 87%) and for 45 of the 48 UniProtKB entries in Experiment II (i.e. 93%).

**Table 9:**
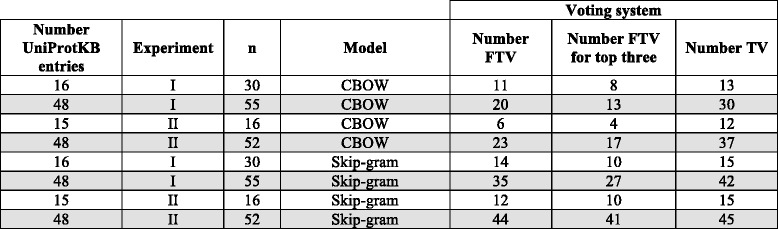
*Performance of CBOW and Skip-gram - Experiment I and Experiment II*: Number of unique UniProtKB entries mapped to CVDO gene and protein classes that participated in Experiment I or II

The rows with grey background remark the 48 UniProtKB entries that participate in both Experiment I and II. Each row of the third column contains the number of target terms for the experiment taking into account the number of UniProtKB entries. According to the voting system, for each model and experiment, the last three columns show: the number of full term variants among the top twelve ranked candidate terms for the UniProtKB entries (Number FTV column); the number of full term variants among the top three ranked candidate terms for the UniProtKB entries (Number FTV for top three); and the number of term variants (i.e. FTV and/or PTV) among the top twelve ranked candidate terms for the UniProtKB entries (Number TV column)

Tables 10 and 11 display the performance of CBOW and Skip-gram for Experiments I and II respectively according to the voting system and considering the categorisation introduced (see subsection ‘Setup of Experiment I and Experiment II for a gene/protein synonym detection task’) that has been applied to the terms from PubMed abstract/title from the small-annotated corpus (first column) as well as to the target terms (second column). From these two tables, it can be observed:

- In Table 10, corresponding to Experiment I, the higher number of target terms corresponds to the category “*Only gene symbol*” (two rows with a grey background) with a total of 34 target terms. CBOW finds TVs among the top twelve ranked candidate terms (nTV column) for 19 of them (i.e. 56%), while Skip-gram finds TVs among the top twelve ranked candidate terms (nTV column) for 29 of them (i.e. 85%).
- In Table 11, corresponding to Experiment II, the higher number of target terms corresponds to the category “*Gene symbol appears*” (three rows with a grey background) with a total of 53 target terms. CBOW finds TVs among the top twelve ranked candidate terms (nTV column) for 39 of them (i.e. 74%), while Skip-gram finds TVs among the top twelve ranked candidate terms (nTV column) for 50 of them (i.e. 94%).
- Comparing results of the voting system for CBOW and Skip-gram, corresponding to both Experiments I (Table 10) and II (Table 11), Skip-gram always obtains an equal or higher number than CBOW for: FTVs variants among the top twelve ranked candidate terms (nFTV column), FTVs among the top three ranked candidate terms (nFTVr3 column); and TVs (FTVs and/or PTVs) among the top twelve ranked candidate terms (nTV column).

**Table 10:**
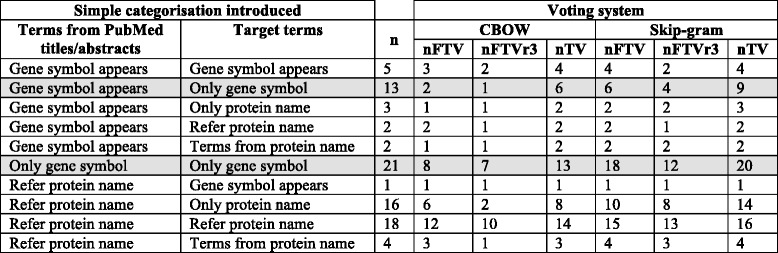
*Results for Experiment I according to the voting system and the simple categorisation introduced*: Results of the voting system according to the simple categorisation introduced (see ‘Setup of Experiment I and Experiment II for a gene/protein synonym detection task’), which has been applied to the terms from PubMed abstract/title from the small-annotated corpus (first column) as well as to the target terms (second column)

Abbreviations: *n* = number of target terms; *nFTV* = number of target terms that have a FTV among the top twelve candidate terms; *nFTVr3* = number of target terms that have a FTV among the top three candidate terms; *nTV* = number of target terms that have a TV (i.e. FTV and/or PTV) among the top twelve candidate terms

**Table 11:**
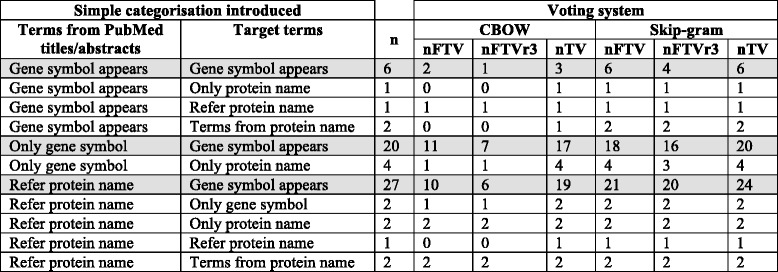
*Results for Experiment II according to the voting system and the simple categorisation introduced*: Results of the voting system according to the simple categorisation introduced (see ‘Setup of Experiment I and Experiment II for a gene/protein synonym detection task’), which has been applied to the terms from PubMed abstract/title from the small-annotated corpus (first column) as well as to the target terms (second column)

Abbreviations: *n* ; number of target terms; *nFTV* ; number of target terms that have a FTV among the top twelve candidate terms; *nFTVr3*; number of target terms that have a FTV among the top three candidate terms; *nTV*; number of target terms that have a TV (i.e. FTV and/or PTV) among the top twelve candidate terms

Table 8 shows, corresponding to both Experiments I and II, the number of FTVs among the top twelve ranked candidate terms (Number FTV column) for Skip-gram is higher than the number of TVs among the top twelve ranked candidate terms (Number TV column) for CBOW. To further illustrate this: a) in Experiment I, CBOW finds 43 TVs while Skip-gram finds 49 FTVs, and b) in Experiment II, CBOW finds 49 TVs while Skip-gram finds 56 FTVs. Tables 10 and 11 provide more details based on the categorisation introduced; it can be observed that for both Experiments I and II, the number of FTVs among the top twelve ranked candidate terms (nFTV column) for Skip-gram is always equal to or greater than the number of TVs among the top twelve ranked candidate terms (nTV column) for CBOW.

We conclude that: a) Skip-gram outperforms CBOW in both Experiments and finds more TVs and FTVs; b) the number of FTVs in both Experiments for Skip-gram is equal to or greater than the number of TVs for CBOW; and c) both Skip-gram and CBOW find more TVs and FTVs in Experiment II (with the aid of the CVDO) than in Experiment I.

## Discussion

The CVDO has a limited lexical content, where each gene and protein class has only one name (i.e. the value of the rdfs:label), and thus lacks term variants (e.g. synonyms and acronyms) for genes/proteins. Keeping the CVDO up-to-date in this respect is a challenge shared with the typical biologist. As Jensen et al. [65] acknowledge that “*for the typical biologist, hands-on literature mining currently means a keyword search in PubMed*”. Both biological entity annotations (gene/protein and organism/species) and molecular interaction annotations (protein-protein and genetic interactions) of the free-text scientific literature are needed to support queries from biologists that may use different names to refer to the same biological entity. However, identification of biological entities within the literature has proven difficult due to term variation and term ambiguity [105], because a biological entity can be expressed by various realisations. A large-scale database such as PubMed contains longer forms including phrases (e.g. “*serum amyloid A-*1 *protein*”) as well as shorter forms such as abbreviations or acronyms (e.g. “*SAA*”). Finding all term variants in text is important to improve the results of information retrieval systems such as PubMed that traditionally rely on keyword-based approaches. Therefore, the number of documents retrieved is prone to change when using acronyms instead of and/or in combination with full terms [106, 107].

This study investigates to what extent word embeddings can contribute to keeping the CVDO up-to-date with new biomedical publications, and furthermore if the CVDO itself can aid such update. Experiment I investigates whether, in taking a gene/protein name from PubMed titles/articles as a target term, it is possible to obtain a list of term variants from the word embeddings created with a 14 M PubMed dataset. The results obtained for Experiment I confirm that it is feasible and that Skip-gram finds 22% more term variants than CBOW using 85 target terms that correspond to 64 UniProtKB entries, which are mapped to CVDO gene and protein classes. Experiment II investigates if the same word embeddings used in Experiment I can produce a better list of term variants (i.e. more term variants) using as target terms a combination of gene/protein names from PubMed titles/abstracts with terms (i.e. more context) from the CVDO protein class expressions and labels. The results obtained for Experiment II show an improvement in performance of CBOW by 11% and Skip-gram by 6% using 68 target terms (fewer target terms than in Experiment I) that corresponds to 63 UniProtKB entries, which are mapped to CVDO gene and protein classes. In Experiment II (with the aid of the CVDO), not only is a better list of gene/protein term variants obtained but also a better ranking, where a full-term variant is likely to appear among the top three ranked candidate terms. Hence, the CVDO supplies context that is effective in inducing term variability whilst reducing ambiguity.

Studies related to semantic similarity and relatedness tasks employ gold standards specific for the biomedical domain that have a relatively small number of term pairs, such as Caviedes and Cimino [38] with 10 term/concept pairs, Pedersen et al. [34] with 30 term/concept pairs, and Pakhomov et al. [36] with 724 term pairs. This study considers a total of 3672 term-pairs from the two experiments together with human judgments from four raters. Hence, an outcome of this study is the creation of a gene/protein names dataset (larger than the MEN Test Collection [40] with 3 K common English word-pairs) that can be reused for the evaluation of semantic models in a gene/protein synonym detection task. However, the overall setup of the two experiments is unbalanced as a result of capturing a realistic scenario where: a) some gene/protein names appearing in PubMed titles/abstracts do not have a vector representation; and b) a gene and its product (typically a protein) can appear together in the scientific text, and thus, the biological knowledge formally represented in the CVDO is already present.

Considering only the 48 UniProtKB entries mapped to CVDO gene and protein classes that participate in both Experiment I and II, the asymmetry between the two experiments can be reduced leading to a smaller gene/protein names dataset with: a) 660 pairs of terms (target term and candidate term) taken from the word embeddings created with CBOW and Skip-gram (i.e. total of 1320 term pairs) and assessed by four raters in Experiment I; and b) 624 pairs of terms taken from the word embeddings created with CBOW and Skip-gram (i.e. a total of 1248 term pairs) and assessed by four raters in Experiment II. Considering only these 2568 term-pairs instead of the total of 3672 term-pairs from the two experiments, the performance obtained for CBOW and Skip-gram is the same as the overall performance reported with Skip-gram outperforming CBOW in both Experiments; and both CBOW and Skip-gram find more term variants in Experiment II (with the aid of the CVDO) than in Experiment I.

Besides the asymmetry between the two experiments presented, there are certain areas of improvement possible regarding the data resources. On one hand, the small-annotated corpus is very narrow in scope with only one curator performing the gene/protein name annotation for 25 PubMed articles (titles and abstracts). On the other hand, the 14 M PubMed dataset used to generate the word embeddings can be arguably larger or include more recent PubMed articles as it only contains titles and available abstracts from PubMed articles published between 2000 and 2016 (files up to 8th June 2016).

As of today, data integration remains a challenge in the life sciences, and therefore, the main curation effort for the sysVASC project is in normalisation. Rebholz-Schuhmann et al. [3] emphasises the lack of a complete solution to normalise proteins and genes (e.g. unique protein identifier together with protein properties and alternative names/labels) that facilitates recognising them from the scientific text. As part of this study, gene/protein names annotated from PubMed titles and/or abstracts are mapped to UniProtKB entries. Other studies have also carried out normalisation whilst making no distinction between genes/proteins. For example, Dogan et al. [108] annotated genes/proteins of interest and manually added their corresponding Entrez Gene identifiers. There are, however, studies that have a list of multiple types of biomedical entities, such as PubTator [109], and BEST [110]. PubTator considers 5 biomedical entities and BEST considers 10 biomedical entities. Both PubTator and BEST perform daily updates of PubMed content and both have automated identification of biomedical entities such as genes. Neither PubTator nor BEST, however, distinguish between proteins and genes.

The results obtained for Experiment II suggest benefits in using target terms belonging to the category “*Gene symbol appears*” introduced – using terms from protein class expressions and labels from the CVDO (or the PxO) – with Skip-gram to automatically obtain the top three ranked candidate terms for a gene/protein of interest. Although this study does not present a tool, it suggests that the CVDO can provide a better context and improve the performance of CBOW and Skip-gram without modifying the word embeddings (i.e. no post-processing of the term vectors is performed), and this could be the foundation for building a tool similar to PubTator or BEST. As the CVDO and the PxO are formalised in OWL, it seems natural to envision a tool based on Semantic Web technologies, such as OWL and SPARQL. Therefore taking into account two annotation properties from SKOS, i.e. *skos:altLabel* and *skos:hiddenLabel*, we can define the automation for the gene/protein synonym detection task as: “*for each CVDO protein, find term variants for the string values within skos:altLabel and store them in skos:hiddenLabel*”.

Levy et al. [29] remarks that if different models “*are allowed to tune a similar set of hyperparameters, their performance is largely comparable*”. The neural language models CBOW and Skip-gram have a similar set of hyperparameters, and thus, their performance has been already compared when accomplishing biomedical tasks [41, 42]. Muneeb et al. [41] applied different hyperparameter configurations and reported a better performance for Skip-gram than CBOW in a semantic similarity and relatedness task for biomedical concepts. Chiu et al. [42] performed a systematic exploration of different hyperparameter configurations and reported an overall better performance for Skip-gram than CBOW in word similarity and NER tasks using biomedical corpora. This study also shows a better performance for Skip-gram than CBOW in a gene/protein synonym detection task considering two metrics: the AUC for the binary classification problem TV and non-TV; and the number of term variants found for 79 UniProtKB entries. We, however, used the same hyperparameter configuration for CBOW and Skip-gram in a study about *patient safety* [111] and it was not possible to determine which (CBOW or Skip-gram) had better performance on an NER task. This study does not exploit Skip-gram with negative sampling, which typically improves its performance [29]. Furthermore, this study does not systematically explore alternative hyperparameter configurations that may lead to performance gains.

As far as we are aware, the use of ontologies to provide more context (i.e. extra terms) for terms selected from the scientific literature has not previously been investigated. This paper demonstrates that the CVDO, and by extension the PxO, can provide better target terms for a gene/protein synonym detection task without altering the word embeddings created by Deep Learning algorithms CBOW and Skip-gram from a 14 M PubMed dataset. At the time of writing BioPortal [112], an open repository of biomedical ontologies, has 551 ontologies. The PxO is re-used by CVDO and is in BioPortal. The experiments reported here can be replicated, and do not demand post-processing of the word embeddings created with CBOW or Skip-gram to obtain performance gains. Therefore, other ontologies from BioPortal may benefit from our proposal to anchor the CVDO in the biomedical literature.

## Conclusion

This study shows performance improvements for both CBOW and Skip-gram on a gene/protein synonym detection task by adding knowledge formalised in the CVDO and without modifying the word embeddings created. Hence, the CVDO supplies context that is effective in inducing term variability for both CBOW and Skip-gram while reducing ambiguity. Skip-gram outperforms CBOW and finds more pertinent term variants for gene/protein names annotated from the scientific literature.

## Additional files

Additional file 1: TermsMapped.xls, this file contains the mapping performed for the 105 terms from 25 PubMed titles/abstracts to 79 UniProtKB identifiers (ACs and IDs) along with the CVDO gene and protein classes labels. (XLS 34 kb)

Additional file 2: CBOW.xls, this file shows the results for CBOW per experiment and rater. (XLSX 175 kb)

Additional file 3: Skip-gram.xls, this file shows the results for Skip-gram per experiment and rater. (XLS 465 kb)

Additional file 4: TermsMapped_votingSystem.xls, this file contains the details of the voting system for CBOW and Skip-gram per experiment. (XLS 70 kb)

Additional file 5: pairwiseIAA.xls, this file contains the values of the Cohen’s Kappa measure for each pair of raters per experiment and model, as well as the average mean. (XLS 8 kb)
